# A Survey of Marker-Less Tracking and Registration Techniques for Health & Environmental Applications to Augmented Reality and Ubiquitous Geospatial Information Systems

**DOI:** 10.3390/s20102997

**Published:** 2020-05-25

**Authors:** Abolghasem Sadeghi-Niaraki, Soo-Mi Choi

**Affiliations:** 1 Geoinformation Tech. Center of Excellence, Faculty of Geodesy& Geomatics Engineering, K. N. Toosi University of Technology, Tehran 19697, Iran; 2 Department of Computer Science and Engineering, Sejong University, Seoul 143-747, Korea; smchoi@sejong.ac.kr

**Keywords:** camera pose estimation and registration, tracking, augmented reality, ubiquitous geospatial information systems, health & environmental applications

## Abstract

Most existing augmented reality (AR) applications are suitable for cases in which only a small number of real world entities are involved, such as superimposing a character on a single surface. In this case, we only need to calculate pose of the camera relative to that surface. However, when an AR health or environmental application involves a one-to-one relationship between an entity in the real-world and the corresponding object in the computer model (geo-referenced object), we need to estimate the pose of the camera in reference to a common coordinate system for better geo-referenced object registration in the real-world. New innovations in developing cheap sensors, computer vision techniques, machine learning, and computing power have helped to develop applications with more precise matching between a real world and a virtual content. AR Tracking techniques can be divided into two subcategories: marker-based and marker-less approaches. This paper provides a comprehensive overview of marker-less registration and tracking techniques and reviews their most important categories in the context of ubiquitous Geospatial Information Systems (GIS) and AR focusing to health and environmental applications. Basic ideas, advantages, and disadvantages, as well as challenges, are discussed for each subcategory of tracking and registration techniques. We need precise enough virtual models of the environment for both calibrations of tracking and visualization. Ubiquitous GISs can play an important role in developing AR in terms of providing seamless and precise spatial data for outdoor (e.g., environmental applications) and indoor (e.g., health applications) environments.

## 1. Introduction

Pose estimation and tracking are the most important parts of ubiquitous GIS-based applications, especially for augmented reality (AR) health & environmental applications. A ubiquitous application supports servicing anytime, supported anywhere and enhanced through technological devices such as AR. There are several techniques available to implement ubiquitous concepts among which AR is one of the most popular ones. To have a precise service in AR, both camera pose and the desired pointing object are required. Consider a simple case in which a 3D character is registered on a surface in the physical environment. To get this, we first need to identify that surface in the physical world. Then, we need to estimate the position of the camera in relation to the surface to be able to register the character on that. This process which is known as camera pose estimation should be done for each frame of the video to track changes in position and update the scene by the 3D virtual model [1].

In the above example, we do not need any previous information about the physical environment. Although we are registering the character to a part of the physical world geometrically, we do not have any semantic link between the entity in the real world and the 3D virtual model. Now, consider a case in which we are going to register the 3D model of a building to the exact location of the corresponding physical entity in the real world. This case involves a one-to-one relation between location of the entity in the physical world and the corresponding object in the computer model which means we need a precise enough 3D model of the environment together with a precise pose estimation and registration (the process of superimposing virtual model to the real world object on the AR display) to augment the virtual model to the physical entity. In this paper, we are mostly focused on these applications where the Geospatial/Geographic Information System (GIS) databases and Building Information Models (BIM) are potentially useful to provide the required 3D model. Many similar purpose applications have been developed by Global Navigation Satellite System (GNSS) receivers and orientation sensors in outdoor environments (e.g., environmental application) but they are subject to several sources of errors which mean they are not enough alone to be used for these applications [2].

The relationship between GIS and AR is not limited to 3D modeling in the tracking and registration process in health & environmental applications. In recent years, GISs have taken advantage of different GIS user interfaces, including line-driven commands, graphic user interfaces (GUIs), virtual environments (VEs), and finally Tangible User Interfaces (TUIs) [3]. Although these interfaces have enhanced the user’s perception of geospatial information, there remains a need for more tangible interfaces to integrate greater public use and increase human interactions with environments and geospatial objects [4]. Therefore, the AR technology can also be another development in the evolution of GIS user interfaces.

In order to develop AR application, especially in the domain of health & environmental applications, in this case, we have several challenges:

- We need a fast method to identify the entity in the real world and refer to the corresponding object in the database.
- We need a precise 3D model of the environment in an offline step.
- We need a precise enough registration method to augment the virtual model to the physical entity.

Pose estimation and tracking benefit several topic domains, including computer vision, sensors, image processing, and information systems. There are several techniques in these domains for tracking and registration. One fast and reliable way to estimate the camera pose is to identify real-world objects by using physical markers embedded in the environment [5]. However, because of some imperfections in these approaches, such as the difficulty in preparing the environment with markers, they are not feasible in large environments such as outdoor settings [6] for environmental applications. Therefore, the more compatible approach which mainly discussed in the paper is to use marker-less techniques to track the camera pose. These methods do not require any other object to be added to the environment.

When the camera pose is estimated, a 3D model of the environment is required to identify which objects the camera is pointing at [2,7]. Then the registration process superimposes virtual information on the target object. For this kind of applications in AR, all entities in the environment should be defined in a common coordinate framework. That means if the environment is a building, then we define all entities in the building in a common coordinate framework. The same is true when the environment is a region or a city especially for environmental applications. 

Because of this diversity in the environments for different AR applications, determining efficient 3D data structures, data storage approaches, formats, and rendering strategies become important [8]. For example, outdoor environments (e.g., environmental application) entail large viewsheds and huge amounts of data, and therefore employing effective strategies to deal with these issues is necessary [9].

Many different approaches have been developed in virtual 3D modeling: computer games, CAD, and geospatial models. Most existing AR studies can be classified into computer games modeling, namely appearance-based approaches encompassing no geographic reference or poor semantically empowered standard formats [10]. The importance of GISs and some CAD formats in tracking and registration derives from the opportunity to develop geo-referenced and semantic-based AR systems, especially for large-scale applications.

In general, AR displays can be divided into two subcategories of see-through and monitor-based displays. AR displays are not in the scope of this paper and we assume all AR applications in this paper to have monitor-based displays in which virtual information is overlaid onto live video frames of mobile devices. As a beginning point for anyone interested in studying display technologies in AR, the survey by Milgram et al. [11] discusses AR displays in a general sense in the context of the reality-virtuality continuum.

Accordingly, the main goal of this paper is to classify and compare all marker-less tracking and registration techniques from the ubiquitous GIS point of view for AR based applications. Existing categories are either general or focus on specific fields. References [12,13] classify them into three subcategories: gravimetric, marker-based, and natural feature-tracking methods. Reference [2] proposed some other categories, including textures, 3D features, and sensor-based tracking. Reference [14] provides a review of marker-less techniques but focus only on vision-based approaches. Schall et al. [15] reviewed sensor- and vision-based techniques simultaneously, but focused only on general categories. Further, none of these studies investigate the role of GISs in AR. In this regard, this paper presents a coherent classification of tracking and registration techniques for mobile AR and provides a detailed review of the marker-less category. Advantages and disadvantages of each subcategory have been discussed and referred to the suitable literature in tables. Also, we did not discuss some other laser-light based distance measurement methods such as Light Detection and Ranging (LIDAR) range finding techniques, which are usually combined with GPS/Inertial Navigation Systems (INS), to provide estimation of camera poses estimation in this paper because the focus was on methods that are cheap and more accessible to the public use. Depth imaging devices such as Kinect were discussed in this paper because they are cheap and their IR technology is popular in many mobile devices.

The rest of this paper is organized as follows: The rest of this section introduces the shift in geospatial information toward a new user interface and discusses the problem of camera pose estimation and tracking and classifies different approaches. Section 2 reviews marker-less technologies including sensor-based and vision-based methods and discusses their advantages and disadvantages. Section 3 discusses the 3D model in AR systems, and Section 4 concludes by addressing issues and open areas for future research.

### 1.1. Evolution of GIS User Interfaces

Over time, geospatial information has evolved from paper maps to desktop GIS, then to web-based GISs, and finally arriving at the current stage of mobile GISs and ubiquitous GIS [16]. The user experience of paper maps occurs completely in the physical world and is restricted to visualizing geospatial/geographic information. With the advent of computers, geospatial information is represented in a virtual space where many difficult and impossible operations in the real world become possible [17]. The first generation of line-driven command GIS user interfaces through which users interact with the computer program with successive lines of text has been replaced with graphic user interfaces (GUIs) and then with 3D virtual environments (VEs) with virtual walking and a bird’s-eye view [3].

**Figure 1:**
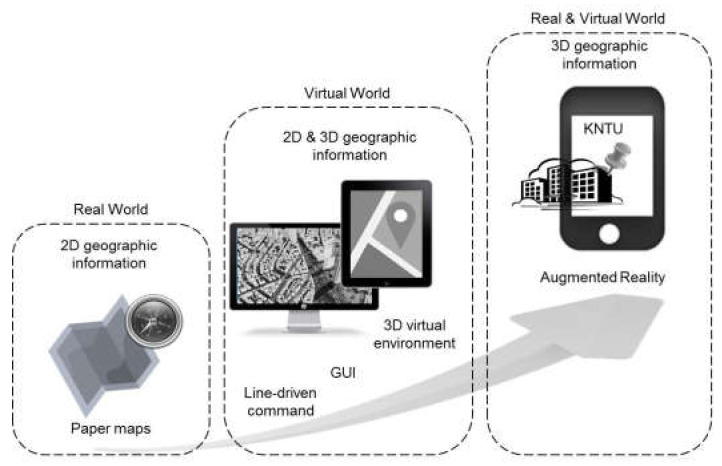
Evolution of GIS user interfaces.

Although these new interfaces increase the user’s perception of geographic information, there remains a need for more tangible interfaces to integrate greater public use and increase human interactions with their environments and physical objects [4]. The advent of technologies such as ubiquitous computing, especially AR, may facilitate a new era of spatial user interfaces in the near future. The key feature of AR in comparison to GUIs and VEs is that the representation of geospatial information and analyses in previous user interfaces takes place completely in the virtual space. However, AR systems integrate the real world with a virtual environment, providing a more tangible experience for the user because of direct interactions with real objects (Figure 1).

### 1.2. AR Tracking and Registration

In AR, the goal is to influence one or more of the human sensory systems such as hearing [18] and vision [19] with virtual information. This paper focuses on influencing the human vision system with virtual information and on enhancing an individual’s view of the real world with computer-generated graphics [1]. Azuma [20] defines AR as follows:

“Augmented reality is a variation of virtual environments (VE), or virtual reality as it is more commonly called. VE technologies completely immerse a user inside a synthetic environment. While immersed, the user cannot see the real world around him. In contrast, AR allows the user to see the real world, with virtual objects superimposed upon or composited with the real world. Therefore, AR supplements reality, rather than completely replacing it”.

Azuma determines that superimposing virtual information on the real world must have three characteristics in AR: (i) It must combine the real and virtual worlds, (ii) it must be interactive on a real-time basis, and (iii) it must be registered in 3D. For this, the first step is to make the interconnection between the real and virtual worlds. In other words, the location of the real objects must first be identified for the camera in 3D and on a real-time basis to augment information on them. This process is known as 3D tracking, which entails the estimation of camera poses of six degrees of freedom (6DOF): three components for the position and three components for the orientation relative to the object [14], 6DOF refers to the object movement on the X, Y and Z axes in 3D space as well as the rotation on pitch, yaw and roll axes. When the pose of the camera estimated in the environment, objects in the camera view can be identified by matching the camera pose to a previously generated 3D model of the environment. Then the graphic elements are registered to the real-world [21]. An accurate, real-time, and robust registration process is one of the most important tasks in AR [22,23].

**Figure 2:**
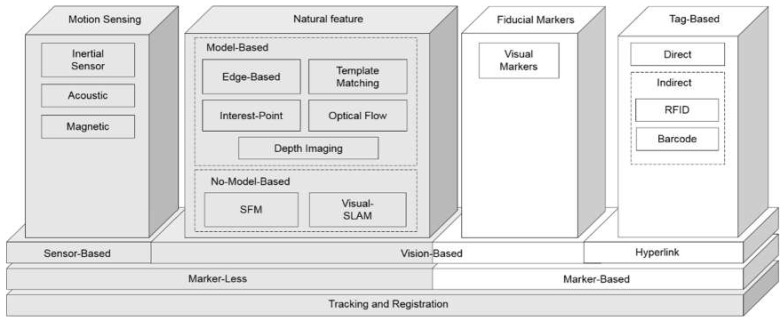
Tracking and registration techniques for mobile augmented reality.

Many approaches have been proposed for tracking and registration in diverse disciplines such as computer vision, image processing, and sensors. This survey organizes the solutions reported in the literature based on whether they need to prepare the environment before use. Therefore, in the very base layer, there are two possible approaches: marker-based and marker-less techniques (Figure 2) which can be used for health and environmental applications. There are several issues needed to be considered to distinguish between marker-based and marker-less based techniques. In marker-based techniques, real-world objects are identified for mobile devices by using physical markers [5]. For this identification process, an explicit image pattern in the AR environment is needed. After that, various registration processes including the creating of a geometry for the positioning of the marker and then superimposing the virtual object on top of it in the real-world scene. Each steps needs several considerations to successfully finish the augmenting process if the camera fails the positioning of the marker then the virtual object cannot be anchored to the real world properly. The marker used in marker-based technique should be anything including lots of corners and edges work especially well, as long as it has adequate exclusive visual features. For the marker-less technique, the geometry needed for superimposing the virtual object on the real-world created by some approaches is based on some software that evokes the environment as virtual model, and placing and positioning to the related scene without dependence to an “anchor” to the real world. In this condition, if the camera loses its line of sight, the virtual model will still be established at the same location. It should be noted that tracking and registration techniques mentioned in Figure 2 also uses for ubiquitous computing-based applications, especially ubiquitous GIS-based applications.

Two subcategories of marker-based techniques include hyperlink and vision-based methods. Hyperlinking links physical objects to web-based content through graphic tags or automatic identification technologies such as the radio frequency identification (RFID) system and contains two subgroups, namely direct and indirect URL discovery methods [24]. Direct methods use active emitters of identifiers, whereas indirect methods use passive devices to provide identifiers for consequent active sensors. Direct methods sense URLs directly from beacons, whereas indirect methods sense identifiers from the physical entity first and then return a URL that bound to that identifier. This URL can provide access to the information related to the entity from the web [25].

1D barcodes, 2D barcodes, and RFID tags are subgroups of indirect methods. RFID tags represent the most popular approach, and near-field communications (NFC) methods have improved their use [24]. Computer vision is an approach with visual markers that define fiducial markers attached to physical objects. In this way, positions of multiple objects are sensed on a real-time basis. A fiducial marker has been used as a point of reference or a measure in imaging systems, which is printed into or on an image while producing the image. Further, additional information such as the orientation, color, size, and shape of objects can be calculated [26].

Marker-based techniques are fast as well as reliable and have potential to be integrated with GIS because they can represent spatial attributes of their location, but they have some drawbacks. There must be uniform lighting and a strong foreground-background contrast for visual markers. In addition, the tracking range is limited by the distinguishability of fiducial markers or tags [14]. Further, it is more difficult to prepare the environment with markers. If vision-based markers are occluded with other objects in a given environment, virtual content cannot be augmented [6]. Markers also require regular maintenance [27]. A comprehensive survey of these technologies has provided in the research by Siltanen et al. [28].

## 2. Marker-Less Techniques

To address the aforementioned limitations, marker-less techniques have been proposed (Figure 2. Grey boxes). Most studies can be divided into two groups at this level: sensor- and vision-based techniques. In both approaches, which can be used for any applications especially for health and environmental applications, the camera pose is the key parameter in connecting real and virtual worlds [29]. In sensor-based techniques, the location and orientation of the camera are determined through positioning methods and sensors [30,31,32]. On the other hand, in vision-based approaches, computer vision and image-processing techniques are employed to estimate the camera pose [14,33,34,35]. It is important to mention that this classification does not separate surveyed approaches into disjoint groups because it is possible for an approach to use both techniques. Table 1 provides the distribution of articles in marker-less techniques.

### 2.1. Sensor-Based Techniques

This section discusses sensor-based techniques and their role in AR applications. In these techniques, sensors and positioning technologies are used to estimate the location and orientation of the camera. A number of popular commercial AR SDKs such as Wikitude (https://www.wikitude.com/) and Layar (https://www.layar.com/) support sensor technologies for camera pose estimation and tracking.

#### 2.1.1. Inertial Sensor Tracking

Inertial sensors reflect a self-contained technology that requires no separate source, which means that they are not limited by other devices such as emitters and cameras [36] Inertial sensors like gyroscopes and accelerometers are embedded in almost all mobile devices.

Gyroscopes use the Coriolis acceleration effect to measure the angular rotation in an inertial space about the input axis and include a rapidly spinning wheel suspended in a housing that resists changes in its orientation [102]. This can be converted into yaw, pitch, and role values and the pose with 3 degrees of freedom. Before any use, raw data must be rectified of any bias (the distance between the data center at zero) and scale (the difference between the range of data from the sensor and real meaningful data) [37].

Accelerometers measure linear acceleration from both the linear movement of the device and the Earth’s gravity [103]. The rotation around the x- (roll) and y- (pitch) axes can be calculated from the accelerometer’s raw data [37]. Because both gyros and accelerometers are affected by errors, they usually fuse to compensate for the weaknesses of each other [104].

Inertial sensors are mostly integrated with positioning techniques such as the GPS in outdoors. Inertial sensors are also suitable for indoor environments but must be integrated with indoor positioning techniques to provide full-camera-pose AR applications.

The integration of an inertial sensor with depth information provided by a depth camera (e.g., Kinect data) helps to improve the visual based pose estimation. This integration procedure overcomes some drawbacks, such as the occlusion. A good example in this regard is the integration of a depth camera with wrist-worn inertial measurement units (IMU) for arm tracking [38]. Reference [39] presents a measurement movement analysis, especially for an indoor health application (arm tracking), using an IMU sensor and its correlation with a depth vision system and an optical fiber sensor. 

#### 2.1.2. Acoustic Tracking

In acoustic tracking systems, ultrasound transmitters and acoustic sensors are used. Ultrasound systems use the time of arrival (ToA) [44], the time difference of arrival (TDoA) [45], and the angle of arrival (AoA) [105] and report localization accuracy in cm. In the ToA method, the user wears ultrasound emitters, and sensors are fixed in the environment [106]. The position and orientation of the device are calculated based on the ToA for sound to reach sensors. Because sound travels slowly, the acoustic tracking system is slower than other sensor-based tracking systems. In addition, the speed of sound in air can vary according to the temperature and humidity of the environment, which can affect the efficiency of the tracking system [13]. Many AoA-based systems use multiple access points (APs) for any target localization. If Aps has large errors, it will lead to a large error for the localization process. To tackle this problem, [46] proposed Unequal AoA Tracking (UAT). This solution utilizes multiple APs and then prioritizes the APs based on their confidence to rule out unreliable measurements. Also, ultrasonic systems based on methods such as TDOA need accurate synchronization between the ultrasound emitters and receivers. Using appropriate synchronization strategy helps to decrease cost and complexity in this case. Reference [47] proposed a method based on using a formula that uses sphere intersection instead of the hyperboloids’ intersection. This method uses TDOA data directly.

#### 2.1.3. Magnetic Tracking 

In a magnetic tracking system, magnetic transmitters and sensors are used. When an electric current is passed through coils (in the source), a magnetic field is created. The position and orientation of receivers are measured relative to the source [3]. Magnetic tracking systems are cheaper to implement but less accurate than other systems [107]. The magnetic field is disturbed in the presence of magnetic materials, such as metal [50]. In addition, magnetic tracking sensors are subject to some jitter and accuracy loss with an increase in the distance and sensitive to electromagnetic noise [51]. Compasses represent the most common magnetic sensor in mobile devices. Magnetic sensors are commonly used in conjunction with inertial sensors to provide a more accurate and stable tracking orientation [2].

Magnetic tracking needs the use of an appropriate method to reduce tracking error for AR markerless process. Using hybrid tracking applying (optical tracking based on optical marker) is a good solution in this regard. Reference [52] proposed a method in which an optical tracking technique is used to decrease magnetic tracking errors. This study also used a high-order polynomial fitting method to correct global errors using continuous mapping and uses smoothing interpolation over the whole measuring space for distal intramedullary nail interlocking for a surgery health application. Reference [53] introduced an electromagnetic tracking solution to overlay 3D virtual images onto the surgical field anatomy obtained by a video camera. For this, there are several challenges that need to be overcome. For instance, tracking markers adapted with a choledochoscope using an electromagnetic field across the patient and sensor coils.

#### 2.1.4. Sensor-Based Tracking and Registration Challenges

There are several sources of errors for sensor-based techniques that can cause a low level of tracking accuracy especially in health and environmental applications. Although these sensors are calibrated before use, they still suffer from accuracy issues. Gyroscope data have inseparable white noise that induces some drift in the rotational angle. This noise accumulates over time and produces inaccurate results [37]. Drift is the most important weakness of gyros and may accumulate up to 10 degrees per minute overtime [102]. Accelerometers measure the acceleration of device caused by the linear movement of the device or the Earth’s gravity field. Separating these two components produces noisy results [108]. To address these errors, data from gyros and accelerometers are usually combined.

Although the nominal accuracy of digital compasses is reported as 0.5 degrees by manufacturers, it can degrade significantly (to six degrees in some cases) with vicinity to cars or other sources of the electromagnetic field [2]. The fusion of the compass with inertial sensors can improve the accuracy of yaw rotation by using its pitch and roll to map magnetometer data to the horizontal plane [37].

Localization with GPS measurements can also be affected by several sources of errors, including atmosphere effects, satellite configurations, and scattering in urban areas [102]. However, some solutions have been suggested to overcome GPS errors, such as the differential GPS network real-time kinematic (NRTK) technique, which enables GPS receivers to localize with centimeter-level accuracy [109]. This accuracy is approximately two times lower for the geodetic height in comparison to horizontal coordinates. The NRTK technique uses different wireless communications area networks to send correction messages calculated in reference stations to the smartphone (rover) [110]. Even in the case of accurate localization, another important issue restricting the registration approach in sensor-based techniques is the accuracy of the GIS model [111].

Galileo, GLONASS, and BeiDou are three other famous Global Navigation Satellite Systems (GNSS) they have many similar characteristics in space and ground segments but at the same time, they use different reference systems and signal structures. Because the numbers of satellites that are contributing in finding the position of a candidate point significantly impact the accuracy of the coordinates, integrating results from these systems is real of interest. Currently, 70 satellites are in the view and when these systems launch all of their satellites this number increase to 120. Integrating observations from these systems need developing new models to exploit the full potential of them [112].

AR displays can enrich many location-based services, although it requires six DoF pose estimation as well as the integration of real and virtual worlds [102]. Here the challenge for AR is the visibility problem [32]. The problem concerns which objects are visible from the user’s point of view to represent virtual information only for them. Visibility analyses are available in the GIS but entail heavy and time-consuming computations [113].

Most position and orientation estimation techniques are theoretically usable in indoor (e.g., health application) or outdoor environments (e.g., environmental application), but some are more compatible with one of the two [114]. Vision-based techniques are less compatible with outdoor AR environments but are more accurate in tracking and registration in comparison to sensor-based approaches. In addition, indoor spaces have received increasing attention because of the huge demand for indoor services. That is, people spend considerable amounts of time in indoor spaces such as shopping malls, metro stations, airports, and hotels, and therefore the use of geospatial information services for indoor spaces is of great importance [58]. In this regard, it is worth reviewing vision-based tracking techniques as one of the most accurate AR approaches with a high level of accuracy and compatibility for indoor environments while considering the role of the GIS and its applications in health and environment filed. Before that, Table 2 summarizes papers regarding different challenges in sensor-based approaches. 

### 2.2. Vision-Based Tracking

In vision-based techniques, images or video sequences of built-in cameras of mobile devices associated with computer vision and image-processing algorithms are used for pose estimation. Most vision-based techniques require a 3D model of the environment for camera pose estimation and tracking, which are called model-based tracking in computer vision [64], but there are also tracking approaches that require no previous information on the environment [84]. We also discuss these methods because they are of great interest in many current AR applications because of their robustness, accuracy, and speed. 

Detectors usually determine the type of model to be used in tracking. With point feature detectors, a database of geo-localized images including 3D point locations and their visual descriptors acquired in an off-line process is used, whereas edge-based systems typically use CAD [33] and GIS [59] models to estimate the camera pose [54].

Vision-based techniques are accurate and reliable, although they are more complicated in comparison to sensor-based approaches [102]. ARKit (https://developer.apple.com/arkit/), ARCore (https://developers.google.com/ar/), and Vuforia (https://www.vuforia.com/) are important AR libraries in vision-based technologies. Vision-based techniques are discussed in the following.

#### 2.2.1. Edge-Based Tracking

Edge-based tracking encompasses projecting a 3D geometric model (GIS or CAD) onto an image and matching it with corresponding edge features of that image. Then the 2D displacement of corresponding features is used to compute the 3D camera motion between frames [60]. Different approaches are used to match a 3D model with edges from images, such as the Marr-Hildreth edge detector, which extracts edges, chains them together, and forms lines to match the 3D model [115], and the Hough transform [116]. A common method is to first render the model and after that apply a sparse 1D search to match the adjacent edges of the model [7,117].

Edge-based tracking is fast and efficient for texture-less scenes, but it is subject to errors caused by background clutter from a large number of local minima. Reference [60] developed a method to address this challenge by partitioning the search space into three levels (interior, contour, and exterior) and local matching of a 3D model to a 2D scene edges. This way they limit their search to only the confident directions that avoid searches across all candidates which decrease the impact of clutter background.

For outdoor urban environments, a common limitation of lines and edges is that single buildings features can be represented. This reduces the robustness of these techniques for dense urban environments. To overcome this problem, Jiao et al. proposed a camera pose estimation system using both a skyline-matching and a GPS method for urban AR applications [61]. Skyline features can model the general geometric characteristic of a street in a geo-tagged image and yield the yaw angle when matched with the skyline extracted from the GIS. To calculate the pitch and role, the system uses a vertical vanishing point technique [118]. The percentage of successful registrations with a rotation error less than 2.0 degrees is 90%, and the average computation time is 671 ms (471 ms for vertical vanishing point detection), which is not sufficient for real-time applications.

3D edge-based tracking is used for rigid objects. This method is categorized in two different techniques: (1) method with explicit edges and (2) method without explicit edges. Within the first method, resemblances between the 3D model edges and the extracted edges are created to retrieve the pose parameters. In the second method, some candidate edges have been selected by searching some strong gradients sampled along the 3D edges near the projections of control points. The first method needs more computation time to detect higher level edge features. This limitation is a big challenge in some devices such as mobile applications. This method is used in specific domain such as tracking polyhedral object (e.g., vehicle and robot arm). One of the advantages of the second method is requiring less computational processes [62].

#### 2.2.2. Interest-Point-Based Tracking

Interest-point-based or point feature methods represent one of the most popular techniques in vision-based approaches. The basic idea of interest-point-based methods is to extract point features from a database of images during an offline training stage and store their locations and visualizing descriptors. Then those feature points extracted from a query image of the camera’s current view are matched to features in the database to estimate the camera pose [70].

Scale-invariant feature transform (SIFT) is a common method for point of interest detection and matching [71]. It is designed to be scale-invariant but is relatively robust across changes in rotation, affine distortion, noise, and illumination, making it capable of matching images with different viewpoints. SIFT can easily extract feature points but is computationally expensive, and therefore many researchers have attempted to improve the performance of this descriptor. For example, the Laplacian/Gaussian feature detector has been replaced with the FAST (Features from Accelerated Segment Test) [119] corner detector, which is faster, but because FAST does not estimate different scales of the feature, it has been reintroduced by storing feature descriptors from all scales [72]. However, interactive frame rates up to 30 Hz are achieved for real-time natural feature tracking. Also, the adapted version of SIFT such as an adaptive scale-invariant feature matching method based on data clustering is proposed to solve the problem of poor robustness during feature matching process [73].

The speeded-up robust feature (SURF) is another interesting point detector/descriptor [74]. It uses the basic Hessian matrix approximation method for detecting interest points and speed up the matching process but does not provide enough speed for real-time applications. For motion tracking SURF, BRISK and AKAZE are alternative for SIFT for motion correction. These detectors are fast and maintain accuracy like SIFT [75].

Reference [76] developed an outdoor-environment method based on coarse GPS localization to restrict the search space in image database features. Then a query to find and match features in the buffer around the position is performed. Here FAST is used to detect key points from images, and the Fast REtinA Keypoint (FREAK) algorithm, a novel keypoint descriptor motivated by the human visual system and MORE which is a faster algorithm to process with lower memory load than SIFT and SURF [120], is used to extract descriptors. Binary descriptors such as FREAK enable fast a run time making for a good solution for real-time application. Binary descriptors have two main characteristics: (1) each bit in the descriptor is independent and (2) uses Hamming distance to estimate similarity measurement instead of Euclidean distance [77]. In addition, RANSAC [121] is used to remove outliers and imperfect matches between features of the query and reference images. However, the proposed system is not as fast as a real-time system and is limited in complex environments. RANSAC is a common method to solve poor stability and multiple mismatching point during image registration. It must be noted that randomness of this method has a negative impact on registration. To solve this problem, an improved SIFT image registration optimization algorithm based on Progressive Sampling Consensus (PROSAC) is a practical solution in this regard [78].

#### 2.2.3. Template Matching

Template-matching techniques employ texture information in images to estimate the camera pose, but unlike interest-point-based techniques, which use features, they take into account a limited area of an image, namely a template, to match reference images usually stored in a database of images in an off-line process and a query image in the current frame of the camera. The match with the best correlation is selected for camera pose estimation [122].

Template-matching approaches are efficient for poorly textured views and applications tracking specific objects in environments such as human body parts [65] and arm robots [66]. An online 3D template-matching algorithm that can reduce the operating time and the amount of data storage has been proposed by Moun et al. [64]. The algorithm uses point cloud data sets with a reduced number of online-built templates and a matching function based on a correlation approach. The algorithm has been evaluated in several different pose conditions for a 78% success rate for matching the computational time of about 7 s. Therefore, the greatest drawback of template-matching approaches is their heavy computation time, which limits their real-time applications.

In fact, using this method needs some improvement steps since template matching gives improper results in cases with limited training samples. Reference [67] proposed a method based on a fast template matching algorithm, which is in turn based on the principal orientation difference feature, to solve this problem. This method uses an extracting edge direction which is divide two parts: 1) the template area based on the position of extracted different features, and 2) searching for the matching position around template. Despite the template matching technique being used in various applications, it suffers from certain drawbacks, such as occlusion. Reference [68] proposed occlusion aware template matching by consensus set maximization to handle these shortcomings since the given results shows good performance.

#### 2.2.4. Optical Flow

Optical flow techniques track a physical point in a video sequence by measuring velocity at each pixel location when projection intensity remains constant [14]. Because images taken at near time, instants are usually closely related to one another. In projecting a 3D path of a moving object onto the image plane, each point produces a 2D path. The instantaneous direction of the path in each point is achieved from 2D velocity. A 2D motion field is provided through 2D velocity for all points visible on the surface. Then the optical method approximates the motion field from time-varying image intensity [81]. Optical based methods are classified in two categories: 1) sparse optical flow and 2) dense optical flow. Sparse optical flow methods, such as Lucas-Kanade, Horn-Schunck, Buxton-Buxton, select important subset of features of objects, and the dense optical flow method, such as the Frneback method, computes optical flow for each pixel. The dense optical flow method weakness is high complexity and execution time, and its advantage is high accuracy and greater depth in comparison to sparse optical flow [82].

Reference [1] proposed a technique based on optical flow to annotate real-world objects with virtual information. Yuan et al. [83] use a simple registration method consisting of two steps. In the first step, four points are specified to build a global coordinate system to superimpose virtual objects on it. Then the Kanade-Lucas-Tomasi (KLT) feature tracker is employed to track natural features in the live video. The optical flow approach is not robust to changes in illumination and large camera displacements, which can cause tracking failure. In addition, this method tends to produce errors because of its sequential pose estimation [122].

#### 2.2.5. Depth Imaging

One of the latest approaches for computing the camera pose is to use depth images containing the distance of scene objects from the camera view as a pixel value. Integrating these depth images and RGB images allow camera pose estimation for tracking [85]. 3D models are often created in offline stages, but depth-sensing allows for model updates and adjustment in real time [86]. In addition, depth information is available through specific hardware such as infra-red (IR) range finders [87] and stereo view algorithms in some mobile devices [88].

Structured light (SL) and time-of-flight (ToF) techniques are two IR-based methods that have recently attracted considerable attention in depth imaging. ToF depth sensors emit waves to target objects and measure for each sensor or pixel the phase delay of reflected IR waves instead of directly measuring the light ToF because of the high speed of light to calculate the distance [89]. It provides a radial range measurement for each pixel and then a transformation between ToF and RGB cameras used to convert Cartesian coordinates of ToF into color camera coordinates [90]. In a structured light system, a 2D pattern is projected onto a target object from an IR projector. Then an IR sensor camera captures the projected pattern distorted because of the object shape and calculates the shift between them by triangulation [91].

Kinect is mainly a gaming IR and RGB imaging device developed through both SL and ToF approaches. The two devices have been compared by La Cascia et al. [92] and found to be more compatible with indoor environments because of the narrow sensor range (3 m for SL and 4.5 m for ToF). Both devices are subject to errors caused by ambient background light, depth inhomogeneity (light reflected from different depths on a pixel for ToF and a lack of depth information because of occlusion for SL Kinect), motion, multipath effects, temperature drift, and a scattering-traveling indirect path for waves. Kinect fusion was the first system to enable camera localization and scene reconstruction in real time. The global model of a scene is reconstructed with camera localization and fusion of dense depth data. The global model is used as a good source to enable estimation of camera pose. This aim is done with aligning the depth map data on to global model. Modified version of ICP is proposed to improve this method. A fast point-to-plane ICP register dense 3D map with global model [93].

#### 2.2.6. No-Model-Based Tracking

An important challenge in any AR and ubiquitous GIS application is when the environment is unknown [94]. The basic idea behind non-model-based techniques is to track and register the camera phone without having a model or database beforehand. Such systems track the motion of the camera and construct a 3D structure of the image scene simultaneously [84,95]. A set of camera parameters includes the focal length, the rotation matrix, and the translation vector, and the camera’s interior parameters may be estimated using the structure from motion (SFM) algorithm in each image. Then triangulation among corresponding points in each image provides an opportunity to compute camera pose [79]. The SFM model does not support real time localization mapping necessary in some health application such as surgical navigation in endoscopy. To solve this problem, the idea of using another interesting approach in non-model-based techniques, which is based on a learning-based descriptor in simultaneous localization and mapping (SLAM) is proposed. This descriptor can be trained using bootstrapping training method [96]. SLAM is conceptualized in robotics based on the idea that it is possible for a mobile robot to move in a completely unknown environment while mapping and localizing simultaneously [97,123]. Consider a mobile robot in an environment with a sensor to take relative observations of some unknown landmarks. If the sensor is a camera applying vision-based observations, then it called visual-SLAM. 

There are many methods for solving the SLAM problem, including probabilistic methods such as the Kalman filter [98] and the extended Kalman filter [84], and geometric approaches such as bundle adjustment [94]. Bundle adjustment is the problem of estimating jointly optimal 3D structure and camera pose parameters through refining a visual reconstruction. To optimize parameters a cost function that quantifies the model fitting error is minimized [124]. Bundle adjustment approaches are more accurate but slower than probabilistic techniques. 

Many attempts have been made to reduce the operating time for the bundle adjustment algorithm. Reference [99] developed an algorithm for estimating the camera motion on a real-time basis and constructing a 3D model of the environment. The authors reduced the operating time by optimizing parameters by a least squares solution. This algorithm uses three images at the beginning to set the global frame and system geometry. Then it uses a robust algorithm for feature detection and matching to compute the camera pose for each frame of the video. A number of frames are selected through a determined process by having key frames incorporated into 3D point triangulation. When a new key frame and 3D points are added, local bundle adjustment is used to simultaneously solve localization and mapping. The processing time for estimating the pose for each frame is about a tenth of a second, but the 3D coordinate mean error is about 0.5 m in comparison to the ground truth.

The accuracy issue is more relevant in outdoor environments with long baselines because this accumulates errors based on frame flow [97]. One way to address this issue is to introduce geo-referenced information to the algorithm. Therefore, some researchers have proposed a post-processing algorithm to add more geometric constraints to correct reconstruction and localization drift by fitting the estimated model with a 3D city model [111]. However, the reconstructed model of the camera is related to the precision of the 3D model. Reference [100] proposed a two-step post-processing algorithm that takes into account the uncertainty of these two models. 

The AR application uses SLAM with another technique, such as Parallel Tracking and Mapping (PTAM), in case that tracking and mapping occurs separately. Some marker-less tracking techniques employ using natural Feature Tracking (NFT) Simultaneous Localization and Mapping (SLAM) [101].

#### 2.2.7. Vision-Based Tracking and Registration Challenges

In addition to common problems associated with accuracy and the operating time, there are some other challenges in vision-based approaches in tracking systems. One disadvantage of some vision-based approaches is their initialization step. Many such algorithms require manual initialization [33,63] or semiautomatic initialization [125]. Even when they initialize automatically, they usually have to start from a well-known point [90]. In addition, when a tracking failure occurs during a fast movement or by dynamic occlusion, their re-initialization is required.

Occlusion is another challenge in vision-based tracking. This occurs when an object is occluded by some part of itself (self-occlusion) or another object (external occlusion). Self-occlusion can be solved by computing a table of visible features [7] or depth buffering [126]. Using outlier detection algorithms such as RANSAC can address external occlusion even in highly cluttered environments with a low inlier information ratio [94]. Table 3 provides more references for handing occlusion in each category.

The ability to provide consistent registration between virtual objects and the real environment is crucial. The phenomenon in which the augmented model is not stable in the scene and oscillates with small amplitudes and high frequencies is called jittering [14]. This may be due to a small number of points available for registration [127].

Major problems in outdoor applications include factors such as weather changes, sunlight, and shadows, which can cause changes in illumination [80]. This weakens vision-based tracking approaches based on intensity information in images (template matching, interest points, and template matching).

In sum, vision-based approaches are suitable for indoor for health applications and outdoor spaces for environmental applications, but because of a high processing cost and huge amounts of required data, they are more compatible with indoor environments. However, they can be used in combination with sensor-based techniques to improve their pose estimation capability. Table 3 provides papers regarding different challenges in sensor-based approaches. Also, a comparison of methods in terms of advantages and disadvantages is summarized in Table 4.

## 3. 3D Modeling

The environment is sometimes limited to only a few objects [129]. In this case, choosing an efficient 3D model is not hard, and almost all existing AR applications belong to this category. However, if the environment is huge (e.g., outdoor environments), then choosing a suitable 3D model for rendering issues becomes a serious challenge.

There are many approaches to virtual 3D modeling: computer games and simulations, CAD, and geospatial/GIS models. In almost all existing AR applications, common 3D games and graphic data formats such as COLLADA, VRML, X3D, and OBJ are used to represent virtual information. In these formats, geometry, material, and appearance are modeled with quite limited topology and semantic information [10]. In CAD approaches, geometry is strongly modeled with limited material modeling. Semantic information and topology are modeled in some particular CAD formats such as IFC, which is used in building information modeling (BIM) [130]. 

GIS data standards such as CityGML model hageometry, topology, and semantic information strongly but with limited appearance [131]. The prominent attribute of GIS data formats that is of great importance, particularly in outdoor AR based environmental applications, is that the GIS is always geo-referenced in any 3D coordinate system [102]. Few studies have used GIS data formats for AR applications. However, to provide a common reference frame for the camera, the use of the GIS is suggested because it can provide not only geometry and semantic modeling but also a solid ground truth to achieve a geo-referenced AR system [8].

Reference [9] extended the application of AR to outdoor scenes with large viewsheds by implementing a client-server Augmented Scene Delivery System (ASDS) for a video webcam at the top of a platform. When the camera rotates, the rendering engine rotates and scales a 3D model to match the camera view. Then the user-selected location UTM coordinates are converted by server into perspective screen coordinates a virtual icon registration onto the captured camera image. The paper employs the TIN data model as the best 3D data structure for large viewsheds because the triangle is the simplest rendering primitive for surface facets in popular rendering libraries such as OpenGL and Direct3D. The results suggest that the linear-time resampling of dense TINs is one of a proper solution for perspective surface rendering.

Reference [132] used a Globe3 Mobile (G3M) framework to render the 3D model of an urban area together with a layer that modelled the solar energy radiation received by each building on images of the physical world captured by the camera of the device. The 3D model was built at different Levels of Detail (LOD) using CityGML standard.

## 4. Critical Discussion

To use the aforementioned comprehensive overview of registration and tracking techniques related to the marker-less approach in above sections for health and environmental applications, we need to consider various and critical challenges and points, especially when there is a smart and ubiquitous environment based on a smart/ubiquitous city. All objects in a smart city are intelligently connected together under an intelligent infrastructure, which was previously mentioned as ubiquitous GIS. AR-based marker-less techniques and mix reality [133] are the most important parts of ubiquitous GIS-based space. Via ubiquitous GIS space, there is a seamless space from both indoor and outdoor spaces. All objects can measure their position from each other. If an Internet of Things (IoT) concept has been implemented in this smart city, then all objected are integrated and can easily perform their spatial analysis using the ubiquitous GIS architectures (e.g., IoT-based AR applications [134]). When we mainly consider AR-based marker-less registration and tracking techniques and services for health and environmental applications in the smart city, several critical discussions and issues will be opened. Since for health and environmental applications, various spaces and disciplines are used, which are intelligently and seamlessly integrated together. Each space has own characteristic, which needs to be related the AR-based marker-less technique. Therefore, we need to use some hybrid techniques to run successful health and environmental applications. In addition to the necessity of the hybrid solution for the critical discussion section, we need also to explain some improvement techniques related to the above mentioned AR-based marker-less techniques since accuracy is a critical point in health (e.g., surgery, etc.) as well as environment (e.g., pollution, etc.) topics, which both are directly related to human welfare. Besides, using more updated approaches related to 3D objects attaching to real objects during the AR augmentation process is the third critical topic to be covered in this critical discussion section.

### 4.1. Hybrid Approach

Regarding the mentioned hybrid technique, it should be noted that many recent studies have introduced various combination methods to deal with AR-based marker-less techniques. As noted above, for complex smart city applications based on ubiquitous GIS space, especially for an AR health & environmental application, the necessity of hybrid techniques are critical. The use of smart health and environmental applications based on AR has an important role in increasing the quality of service to citizens, which is the goal of a smart city. Most smart health and environmental applications are in indoor and outdoor spaces, respectively. Although, the reverse situation is also the case, there are a variety of cases indoors for health applications and environmental applications are usually outdoors and in a wide field. In many of these applications, it is not possible to use a marker in the environment (indoor and outdoor), and therefore the more attention come to marker-less tracking approaches.

During the hybrid process AR marker-less techniques, there are considerable challenges to be addressed. To open those critical challenges in this section, we introduce some examples from recent studies. For instance, Oskiper et al. proposed a hybrid method of both marker-less tracking techniques, including vision based and sensor-based approaches [135]. This research offered an integrated solution for a SLAM based tracking with fusion with an accelerometer and a gyroscope. The aforementioned SLAM is a no-model based of the vision-based tracking, in which there are non-existing models and environments during the tracking step. In a hybrid approach, the integration of sensor information with SLAM will be very helpful. For some health and environmental mobile applications, the current marker-less tracking techniques need a hybrid solution to deal with some resource management limitations in the mobile environment. Using the SLAM method for large scale mobile application encounters mobile resource limitations. A hybrid study is introduced for SLAM and other sensor-based solutions in Correa et al. [136]. Also, Park presented a hybrid structure for mobile smart devices for a marker-less tracking-based image registration using natural features and a third person perspective augmented view [137]. This system employs augmented reality to enable designation of thermographic targets in a façade inspection task.

There are some other types of a hybrid solution between a marker based and a marker-less tracking techniques. Reference [138] discussed this sort of hybrid approach. One health-related application of such a hybrid solution is used for rehabilitation training, including movement analysis. Reference [139] proposed a novel hybrid tracking for the rehabilitation training employing a custom-made colored marker-based tracking and a vision based marker-less tracking technique using Kinect. Reference [138] discussed a hybrid solution using a marker-less method (using a CAD model) and a marker-based method (using images) in the field of cultural heritage visualisation which has environmental constraints. In this scenario, there are various limitations to using one marker-less tracking, such as the existence of difficulty applying only an edge detection method using the CAD model for a damaged section of a cultural heritage site. For this condition, due to a light contrast variation, marker identification detection will be difficult in outdoor areas. An image-based marker solution will be used as hybrid approach to tackle this shortcoming.

There is another possibility of a hybrid solution inside a specific marker-less or marker-based tracking solution. For instance, Kim et al. explained hybrid techniques, including various algorithms used for each step of a marker-less tracking solution including: Gaussian filter for better visualization quality in a pre-processing stage, GrapCut algorithm for high accuracy for object segmentation during live video stream in a segmentation stage, Iterative Closest Point algorithm for straightforward object identification in a feature extraction stage [140].

We can consider a hybrid solution simultaneously referring to the integration of multiuser tracking techniques usage. This can be very popular in future trends of AR-based tracking solutions for many applications, especially for health and environmental applications. Reference [128] tried to explain a prominent idea to use multiuser head tracking using multi camera based on the voxelization of dense point cloud data. This method defined elements to be tracked using segmentation and then uses dedicated Particle Filter for user tracking. For such multiuser based hybrid methodology for outdoor environmental applications, handling multiple users using multi-cameras tracking to make a secure interface for increasing users’ interaction is critical issue. Reference [141] developed a prototype called DataCube, which enables 3D data visualisation and manipulation with supporting multiuser in 3D space. This prototype enables users’ gesture control interaction using appropriate filtering strategy to manage data size. This mechanism uses a security method to enable user visibility during interaction and shows their feedback to each other with facial reactions.

### 4.2. Improvement Approaches

Many other improvment approaches for marker-less techniques have been introduced in addition to the hybrid strategy, which can be used for a smart environment, especially for smart-based health and environmental applications. For smart health applications Hu et al. proposed a deep learning approach using a fully convolutional neural network for tumor tracking in stereotactic lung radiotherapy to improve the existing marker-less technique [142]. This method used personalized training data sets obtained from patients to handle real-time tracking during surgery. Besides, Caron et al. improved marker-less registration and tracking method based on depth imaging and deep neural network for a bone surgery smart health application [143]. In this method, a depth camera obtains RGB, and the depth image of bone and deep neural network are used for localizing and segmenting the surgical target. For smart environmental applications, Dame et al. suggested an improving tracking tool using a Python deep learning-based pose estimation for animal tracking, which is useful for environmental studies such as monitoring species at risk of extinction [144]. This application uses active-learning-based network refinement for pose estimation, which provided suitable results in the case of limited training datasets.

Other studies have introduced some additional techniques for improving the current vision- based marker-less tracking methods. Reference [145] presented a fast corner detection method, which used a user defined target and extended tracking to improve the SLAM method result using ray cast to create labelling of the objects in the scanned object for multi-various intensities applications.

Reference [146] improved the speed of the SURF marker-less tracking method using Binary Robust Invariant Scalable Key-points (BRISK), which is the scale and rotation invariant binary descriptor. Finally, there other issues which should be considered for the improvement of AR-based tracking and registration processes. Some studies tried to introduce some solutions for the augmentation improving process, especially for health and environmental applications. Reference [147] presented a smart health application for when a patient moves during a sensitive surgery operation since such body movement leads to considerable disruption during the tracking process for during tracking and registration for the AR application.

### 4.3. 3D Object Modelling Approach

There are other AR-based tracking techniques with respect to augmenting 3D virtual objects to the real world, considering several issues such as 3D virtual objects pose estimation and camera localization. Reference [148] presented two different categories, including coplanar based techniques and available 3D model methods, for 3D pose estimation. The first category contains two subclasses, geometric and the appearance-based method. The second one includes two subclasses, on-line estimated 3D model (e.g., SLAM, V-SLAM, etc.) and available 3D model methods. Reference [149] proposed a 3D-AR marker-less image registration method using a stereo matching algorithm, applying the patient’s CT-derived 3D model and an iterative closest point method for maxillofacial surgery in the field of smart health application. This study solves misalignment challenge during registration with using fiducial mark attaching on patient body and stereo camera space.

## 5. Conclusions and Future Trends

The literature review offers some important insights. The focus of this paper was on the applications of marker-less AR, in which the ubiquitous GIS-based environment entities are defined in a common coordinate framework. This includes both indoor (e.g., health) and outdoor (environmental) applications for AR. Our goal was to review the literature on pose estimation for registration and tracking to discover which approaches are potentially more suitable for this purpose. A one-to-one relation between the real-world entities and the virtual model requires a 3D model of the ubiquitous GIS environment. 

Sensor-based and positioning techniques provide very coarse pose estimations for AR and ubiquitous GIS. Although they are very simple, with widespread use, computationally inexpensive, and provide a common reference system together with a 3D model, they are insufficient for applications that need precise tracking and registration [150,151]. However, their potential to be used as an initial coarse pose estimation method has been discussed in many papers.

On the other hand, vision-based methods are very diverse and can provide more reliable and accurate pose estimation and be tracking although they are computationally expensive. One important challenge in these methods for the specific purpose of this paper is that they are not easy to define in a common coordinate system with the 3D model. Most of these methods work based on extracting features from the image which then need to be matched with the previously built model to fulfill the goal of transforming to the 3D model coordinate system. This transformation usually needs a precise and reliable manual or semi-automatic initialization which is also restricted by 3D model accuracy. Edge-based methods are potentially more applicable than interest point based and optical flow methods to be matched with GIS to automatize the initialization because they can benefit from similarity measure between a shape of the lines on an image and the 3D model. Template matching methods are less applicable to AR applications discussed in this paper. Very popular No-model based techniques usually use one of the previous approaches for pose estimation. Their strength is to provide accurate, reliable, and fast pose estimation and mapping but they are also challenging to be used for the purpose of this paper.

Calibrating methods with a 3D model is not limited to the initialization process but most of the techniques need to be matched to the 3D model again especially in long baselines. The processing time and computing resources of AR marker-less tracking needs more future studies since these are still very challenging making it difficult to create online health and environmental applications especially in this case that we have large environment and models. Further research is needed to evaluate the pose estimation uncertainty as well as computing time and resources for large environments and models for marker-less techniques.

As explained in our presentation of various new marker-less trends in the critical discussion section, they required massive anticipated studies considering hybrid, improved and 3D augmented objects for AR-based applications, especially for indoor health and outdoor environmental applications for new emerging trends such as smart cities, ubiquitous spaces and IoT-based architecture. For instance, emerging IoT-based AR applications needs more study to propose novel methods and interfaces to handle the integration of the tracking mode using IoT sensors. Handling complex conditions such as a complex objects, sudden motion during registration, and tracking is a considerable challenge requiring further study. Using adaptive methods for resource management of mobile applications is a good research area for future study. The development of new AR health and environmental applications such as AR-drone-based application needs further study for a fast non-model-based method. These applications provide remote control functionality, which is significant for emergency applications. With respect to hybrid approaches, combining marker-less tracking and new trends such as deep learning and blockchain are suggested for further research. These approaches need to develop novel deep and secure chain network structures to handle large amounts of data to support real-time health and environmental applications.

## Figures and Tables

**Table 1:** Distribution of papers in different categories.

Classification Criteria	References
1. Marker-Less	
1.1. Sensor-Based	
1.1.1. Inertial	[2,15,36,37,38,39,40,41,42,43,44,45,46,47,48,49]
1.1.2. Acoustic	[50,51,52,53,54,55,56,57]
1.1.3. Magnetic	
1.2. Vision-Based	[7,33,54,58,59,60,61,62,63]
1.2.1. Model-Based	
1.2.1.1. Edge-Based	[64,65,66,67,68,69,70,71,72,73,74,75,76,77,78,79,80]
1.2.1.2. Template matching	[1,63,81,82,83]
1.2.1.3. Interest-Point- Based	[84,85,86,87,88,89,90,91,92,93,94,95,96,97,98,99,100,101]
1.2.1.4. Optical flow	
1.2.1.5. Depth imaging	
1.2.2. No-Model-Based	

**Table 2:** Challenges in sensor-based techniques.

Challenges.	Inertial	Acoustic	Magnetic
Accuracy	[40,41,42]	[44,45,48]	[51,54,55,56,57],
Drift	[37,40,41]	[44,48]	[10,50,55],
Visibility		[44]	[32]
GIS model	[2,15]	[106]	[19]
Indoor	[41,42]	[44,48,49]	[50,56]
Outdoor	[15,40,42],	[48]	[10,29]

**Table 3:** Challenges in vision-based techniques.

Challenges	Edge-Based	Template Matching	Interest Point	Optical Flow	Depth Imaging	No-Model-Based
Automatic initialization	[58]	[64,65,69]	[72]	[1]	[88,89]	[84,99]
Manual initialization	[33,54,59,63]		[80]	[63],	[85]	[95,100]
Occlusion handling	[33,54,60,63]	[65,66,69]	[8,72]	[1,63,81]	[85,87,88,89]	[84,94,95]
Jitter	[33]		[72,118],	[83]		[84]
Handling illumination changes	[33,60,63]	[69]	[71,76,79,80],	[63,81,83],	[90,92]	[94]
Compatible with GIS environments	[58,59,61]					[100,111]
Compatible with CAD environments	[7,33,54]	[64]				
Indoor	[7,33,60]	[65,66]	[128]	[1,83]	[89,92]	[84,97]
Outdoor	[54,58,59,61],	[69]	[60,64]	[1,83]	[85,90]	[59,84,97,99,111]

**Table 4:** Summary of advantages and disadvantages of pose estimation and methods.

Category			Device/Algorithm/Method	Advantage	Disadvantage
Sensor-based		Inertial	Gyroscope,	Self-contained, popular in mobile devices, fusion possible to overcome errors, applicable to indoor/outdoor, real-time	Bias & rectification required, gyros have inseparable white noise, accumulate errors, drift up to 10 degrees/min, need positioning systems
			Accelerometer		
		Acoustic	ToA, TDoA, AoA	6 DoF pose estimation	Sound travels slowly, sensitive to environment (humid, temp, etc.), not popular in mobile devices
		Magnetic	Compass	3 DoF (orientations) & 6 DoF (not popular) pose estimation possible, real-time	Less accurate than inertial methods, subject to magnetic field distortion & jitter, need positioning systems in case of 3 DoF, error up to 6 degrees
Vision-Based	Model based	Edge-Based	Mar-Hilldreth edge detector,	Compatible with GIS/CAD models, excellent for texture-less objects, applicable to indoor/outdoor, very reliable, automatic initialization possible	Background clutter errors, not fast enough for real-time applications, rotation error about 2 degrees, position error 10–15 cm
			Hough transform		
		Interest Point Based	SIFT, SURF, FAST, RANSAC, FREAK	very reliable in feature extraction (scale, orientation, affine transformation, and illumination invariant), very accurate registration, applicable to indoor/outdoor	Mostly compatible with point clouds & image databases, initialization to GIS models is challenging
		Template Matching		Efficient for poorly textured views, automatic initialization, applicable to indoor/outdoor	Heavy computation time, not applicable to vector based GIS,
		Optical Flow	KLT	Useful for tracking movement, applicable to indoor/outdoor	Not robust to illumination change & large camera displacement, cumulative error
		Depth imaging	Structured Light (SL), Time of Flight (ToF)	IR sensors are becoming popular in mobile devices, applicable to indoor/outdoor	Narrow sensor range (SL, 3 m; ToA, 4 m), subject to errors caused by ambient background light, depth inhomogeneity, motion, multi-path effects, and temperature drift
	No-Model Based		SFM, Visual-SLAM, bundle, KF, EKF	Very popular, useful for applications in unknown environments, applicable to indoor/outdoor	Initialization and matching to a reference mode is not easy, accumulate error,
